# Hypothetical protein FoDbp40 influences the growth and virulence of *Fusarium oxysporum* by regulating the expression of isocitrate lyase

**DOI:** 10.3389/fmicb.2022.1050637

**Published:** 2022-11-28

**Authors:** Busi Zhao, Dan He, Song Gao, Yan Zhang, Li Wang

**Affiliations:** ^1^Department of Pathogenobiology, Jilin University Mycology Research Center, Key Laboratory of Zoonosis Research, Ministry of Education, College of Basic Medical Sciences, Jilin University, Changchun, China; ^2^Beijing ZhongKaiTianCheng Bio-technology Co. Ltd., Beijing, China

**Keywords:** *Fusarium oxysporum*, CCCH-type zinc finger, ICL, growth, AMPK/mTOR

## Abstract

Fungal growth is closely related to virulence. Finding the key genes and pathways that regulate growth can help elucidate the regulatory mechanisms of fungal growth and virulence in efforts to locate new drug targets. *Fusarium oxysporum* is an important plant pathogen and human opportunistic pathogen that has research value in agricultural and medicinal fields. A mutant of *F. oxysporum* with reduced growth was obtained by *Agrobacterium tumefaciens*-mediated transformation, the transferred DNA (T-DNA) interrupted gene in this mutant coded a hypothetical protein that we named FoDbp40. FoDbp40 has an unknown function, but we chose to explore its possible functions as it may play a role in fungal growth regulatory mechanisms. Results showed that *F. oxysporum* growth and virulence decreased after FoDbp40 deletion. FOXG_05529 (NCBI Gene ID, isocitrate lyase, ICL) was identified as a key gene that involved in the reduced growth of this mutant. Deletion of FoDbp40 results in a decrease of more than 80% in ICL expression and activity, succinate level, and energy level, plus a decrease in phosphorylated mammalian target of rapamycin level and an increase in phosphorylated 5′-adenosine monophosphate activated protein kinase level. In summary, our study found that the FoDbp40 regulates the expression of ICL at a transcriptional level and affects energy levels and downstream related pathways, thereby regulating the growth and virulence of *F. oxysporum*.

## Introduction

*Fusarium* species belong to a large genus of filamentous fungi which can infect plants and humans. In 2022, *Fusarium* species were incorporated in High Priority Group of the WHO fungal priority pathogens list (WHO, 2022). *Fusarium oxysporum* can infect cotton, rice, wheat, and other crops, causing diseases including cotton wilt, crown rot in cereal crops, and head blight in wheat. The fungus seriously affects food safety and causes huge economic loss (Kazan and Gardiner, 2018; Melotto et al., 2020; Zhu et al., 2021). *Fusarium* species are also important opportunistic pathogens in humans, mainly causing corneal infection (keratitis; Alkatan and Al-Essa, 2019), but it can also cause invasive and disseminated infection in immunocompromised people, posing threats to human health (Nucci and Anaissie, 2007; Muraosa et al., 2017).

Fungi virulence is closely related to growth. The cell wall is an essential structure for fungal growth, and some components of cell wall, such as β-1,3-glucans, can participate in activating host immune response and affect virulence in *Aspergillus fumigatus* (Chotirmall et al., 2014). In *Cryptococcus neoformans*, a microtubule-associated CAP-glycine protein (Cgp1) can promote the production of capsules, thereby enhancing virulence (Wang et al., 2018). In *Aspergillus fumigatus*, *Beauveria bassiana*, and *Fusarium graminearum*, strains with slowed growth and sporulation were found to have lower virulence (Paisley et al., 2005; Safavi et al., 2007; Wang et al., 2021). Therefore, studying and finding key regulatory factors, mechanisms, and pathways that regulate fungal growth is crucial to understanding the fungal growth profile, finding new drug targets, and fungal prevention and control methodologies.

Hypothetical proteins are proteins predicted to be expressed from an open reading frame, but with unknown function. Presently, scores of hypothetical proteins exist in the genomes of various animals, plants, fungi, and microorganisms, potentially involving diverse biological processes, including gene expression and protein folding, as well as various life functions, such as host-pathogen interactions and drug tolerance (Wang et al., 2012; Uddin et al., 2019; Pranavathiyani et al., 2020). Hypothetical proteins that regulate growth and virulence have been found in the bacteria *Chlamydia trachomatis* and fungi *Magnaporthe grisea*, among many others (Chen et al., 2006; Lin et al., 2021).

The glyoxylate metabolism pathway is an anabolic variation of the TCA cycle that occurs in most other organisms and converts isocitrate to glyoxylate and succinate. It plays an important role in the growth, pathogenesis, and stress tolerance of fungi such as yeast and *Fusarium* species (Park et al., 2016; Vico et al., 2021). ICL is a key enzyme in the glyoxylate metabolism pathway, responsible for catalyzing the synthesis of succinate, thereby regulating carbon metabolism and ATP synthesis (Gengenbacher et al., 2010; Selinski and Scheibe, 2014). ICL is not present in human and is, therefore, a potential therapeutic target against fungal infection (Bhusal et al., 2017).

Nucleic acid-binding proteins can bind to specific sequences of DNA or RNA and are involved in the transcriptional regulation of cellular processes, such as DNA damage repair and gene expression (Andres et al., 2019; Bartas et al., 2021). Zinc finger proteins are the most abundant class of transcription factors in eukaryotic genomes. These proteins can bind to DNA or RNA, even protein to regulate transcription and play an important role in many life processes (Corkins et al., 2013; Zou et al., 2018). According to protein sequence, fold, and function, zinc finger proteins can be divided into over 20 primary types, such as C_2_H_2_, CCHC, CCCH and so on. Current research mainly focuses on C_2_H_2_ type zinc finger proteins (Interpro IPR036236), which have the zinc ion coordinated by two cysteine and two histidine residues. CCCH-type zinc finger proteins have a zinc ion coordinated by three cysteines and a single histidine (C-x8-C-x5-C-x3-H; Interpro IPR036855) and account for about 0.8% of zinc finger proteins (Berg and Shi, 1996), but have seldom been reported in fungi. CCCH zinc finger proteins are known as RNA-binding proteins and associated with post-transcriptional regulation of mRNA (Fu and Blackshear, 2017). In addition to its role in RNA metabolism, recent studies demonstrated that CCCH zinc finger proteins also modulate transcription (Zou et al., 2018; Wang et al., 2022). A RNA-binding CCCH zinc finger protein Zc3h10 was also proved to activate UCP1 promoter by binding to a distal upstream region (Yi et al., 2019).

In this study, a mutant of *F. oxysporum* with reduced growth was obtained by *Agrobacterium tumefaciens*-mediated transformation (ATMT), in which the expression of main genes involved in glyoxylate metabolism pathway were down-regulated. The T-DNA interrupted gene FOXG_12762 encodes a hypothetical protein containing CCCH-type zinc finger--FoDbp40 [Fo for *F. oxysporum*, Dbp for DNA binding protein, 40 (kDa) for the calculated molecular mass]. The regulation of the expression of ICL by FoDbp40 was elucidated, and the influence of FoDbp40 on the growth and virulence of *F. oxysporum* was discussed.

## Materials and methods

### Construction of random insertion *Fusarium oxysporum* mutants

Wild type *F. oxysporum* JLCC31768 and *Agrobacterium tumefaciens* AgrN (containing plasmid pXEN carrying neomycin and kanamycin resistance tags[*neo*]) were used to generate *F. oxysporum* mutants (He et al., 2021). Wild type and AgrN (Table 1) were preserved at and obtained from the Jilin University Mycology Research Center (Jilin, China).

**Table 1:** Strains used in this study.

Strain name	Information
WT	Wild type of *Fusarium oxysporum* JLCC31768, which was obtained from the Jilin University Mycology Research Center (He et al., 2021)
FOM312	T-DNA inserted mutant with reduced growth and virulence (obtained in this study)
*Δ*12762	Deleted FOXG_12762 from wild type (obtained in this study)
C12762	Complemented FOXG_12762 to *Δ*12762 (obtained in this study, EGFP contained)
AgrN	*Agrobacterium tumefaciens* containing pXEN, which was obtained from the Jilin University Mycology Research Center (He et al., 2021)

ATMT of *F. oxysporum* was performed as described previously to obtain mutants with single-strand transferred DNA (T-DNA) inserts (He et al., 2021). The DNA of randomly selected mutants containing the *neo* gene was isolated and amplified using DNA extraction kits (Beyotim, Shanghai, China) and specific neoF and neoR primers (He et al., 2021). The products were then sequenced by Comate Bioscience Co., Ltd. (Jilin, China) to confirm whether T-DNA was inserted into the *F. oxysporum* genome.

### Analysis of T-DNA interrupted gene

Sequences flanking the inserted T-DNA were amplified by touchdown thermal asymmetric interlaced polymerase chain reaction (TAIL-PCR) using previously described primers (Gao et al., 2016). The products were sequenced (Comate Bioscience Co., Ltd. Jilin, China) and aligned against the *F. oxysporum* f. sp. *lycopersici* genome (GCF_000149955.1) using the Basic Local Alignment Search Tool (BLAST^1^) to determine the insertion sites (Lorenzini and Zapparoli, 2019). Bioinformatic analysis for nuclear localization signals was performed by NLStradamus program (Cheng et al., 2019; http://www.moseslab.csb.utoronto.ca/software/).

### Construction of the FoDbp40 deletion and complementation strain

We based the method for constructing the targeted knockout of FoDbp40 on homologous genetic recombination by ATMT, with the *neo* marker gene replacing the target gene (He et al., 2021). Primers used are listed in Supplementary Table S2.

Our complementation strain was constructed according to methods described earlier (Roth and Chilvers, 2019). The FoDbp40 open reading frame and its own terminator region were amplified separately. To visualize the localization of FoDbp40 in *F. oxysporum*, the enhanced green fluorescent protein (EGFP) open reading frame was amplified from pEGFP-N3 by PCR. The resulting three DNA fragments were ligated using a One Step Cloning kit (Vazyme, Nanjing, China) and the resulting construct was transformed into protoplasts to create the deletion mutant we named *Δ*12762. The verification of the deletion and complementation were completed by PCR and observed phenotype. Primers used for all reactions are listed in Table 2. Graphs showing mechanism of the methods were also provided in Supplementary Figures S1, S2.

**Table 2:** Primers used for the vector construction.

Name	Sequence	
12762LF	GATCTTCACTAGTGGGAATTCAGGGCCGCAACGGAAAC	PCR primers for the construction of the gene deletion
12762LR	AGCTCGAATTGCAAGGAGGGAGCGTCAAAGAA	
12762RF	CAGAATAAAGTTTGAGGTCCTGGTGGTGGT	
12762RR	CAGGTCGACTCTAGAGGATCCACCCGTTGCAGTCAAAGCC	
12762NF	CCCTCCTTGCAATTCGAGCTCGGTACCCAG	
12762NR	GGACCTCAAACTTTATTCTGTCTTTTTATTGCCGTCCC	
12762p-F	gaccatgattacgccaagcttGCTGAGAAGGACAGGCCG	PCR primers for the construction of the complementation vector
12762p-R	ttacccttcttgggaggcatGATGGGCAGTTGGTGGCG	
12762 + e-F	ATGCCTCCCAAGAAGGGTAAG	
12762 + e-R	cTTACTTGTACAGCTCGTCCATGC	
12F	CCGCTAGCGCTACCGGACTCAGATCTATGCCTCCCAAGAAGGGTAAGGAGG	PCR primers for construction of the vector for Luciferase reporter assay
12R	GCGATGGATCCCGGGCCCGCGGCCGCTGCCGCCGCCGCCGCTGCCGCCGCCGCCAGATCCGGTTGCTGTCTCAGCTA	
05529proF	ctggcctaactggccggtaccCACAGAGGAAGCAGAGCGAATT	
05529proR	cagtaccggattgccaagcttTCTAGCTCGGCTTTCCACCG	
XF	CGAGTGGTGATTTTGTGCCG	PCR primers for identification
XR	AAACTGAAGGCGGGAAACGA	
TRF	GCCTATGGAAAAACGCCAGC	
TRR	CAACTGTTGGGAAGGGCGA	

According to the instruction, some primers for vector construction was design with a cohesive end which was wrote as normal letter.

### Growth analysis and microscopic examination

*Fusarium oxysporum* was grown on potato dextrose agar (PDA) for 5 days at 25°C for growth analysis. The conidia were washed down with sterile 0.85% saline containing approximately 1%—Tween® 20 and diluted to 1 × 10^5^ CFU/ml. Then 2 μl of the suspension was dripped onto PDA plates and grown for 5 days at 25°C. Conidia were collected from 5-day-old cultures on PDA. The quantification for each strain was performed in triplicate. Each plate was washed three times with sterile 0.85% saline containing approximately 1%—Tween® 20 and the conidia suspension were adjusted to appropriate volume.

Slide cultures were prepared and then examined with microscope after lactophenol cotton blue staining. To visualize the localization of FoDbp40, the slide cultures were stained with 10 μg/ml 4′,6-diamidino-2-phenylindole (DAPI; Beyotime, Jiangsu, China) for nuclei staining, and then examined with a BX53 microscope (Evident Olympus, Tokyo, Japan).

### Virulence assay

Human corneal epithelial cells (HCEC) were purchased from BeNa culture collection (Jiangsu, China), maintained in Minimum Essential Medium (MEM; XP Biomed Ltd., Shanghai, China) supplemented with 10% heat-inactivated fetal bovine serum (FBS; Gibco, New York, NY, USA) and cultured in 60 ml flasks kept at 37°C in a humidified incubator containing 5% CO_2_.

For *in vitro* cytotoxicity assay, the cultured HCEC were co-cultured with *F. oxysporum* conidia for 24 h (Kolar et al., 2017) in 96-well plates (1 × 10^4^ cells/well), then the lactate dehydrogenase (LDH) released from the cultured HCEC was measured using a lactate dehydrogenase cytotoxicity assay kit (Beyotime, Jiangsu, China; Jin et al., 2007).

An *in vivo* virulence assay was performed with AB strain zebrafish (ZFIN ID: ZDB-GENO-960809–7) as previously described (Laanto et al., 2012). Briefly, zebrafish (three-day post-fertilization) were infected with the *F. oxysporum* conidia by bathing. The fish were individually challenged with 1 × 10^4^ CFU/ml conidia, and survival was recorded every 12 h. All the experiments in this study were approved by the animal ethics committee of Jilin University.

### Analysis of gene expression by RT-qPCR

The RNA extraction and construction of cDNA libraries was performed as described previously (He et al., 2021; Wei et al., 2022). Conidia of *F. oxysporum* (1 × 10^6^ CFU) were added to PDB medium and incubated for 24 h. The mycelia were collected and ground to a powder in liquid nitrogen. Total RNA was extracted from the ground material using RNAiso Plus (TaKaRa, Japan). Real-time, quantitative PCR (RT-qPCR) analysis was performed with a SYBR Green master mix (Monad, Shanghai, China) and the ABI QuantStudio 3 PCR system (Applied Biosystems, Waltham, MA, USA). Relative expression levels of the genes were calculated using the threshold cycle (2^−ΔΔCT^ also known as 22DDCT) method (Livak and Schmittgen, 2001). Gene expression levels were normalized against the expression of the 18S rRNA housekeeping gene (Table 3). Details regarding the relevant primers are provided in Supplementary Table S3.

**Table 3:** Primers used for qPCR.

Name	Sequence
Fu18SF	CGCCAGAGGACCCCTAAAC
Fu18SR	ATCGATGCCAGAACCAAGAGA
05529F	GAAGGAGGTTGAGGCTGTCAAG
05529R	CGTAGGTGTAGCTGGCATCTC
10116F	AGCTCTGATGGTCCCTGGAT
10116R	TGCGTTTACAACCAGAAAGCAG
01304F	ACCTAAGCGAAACGGGTCTG
01304R	ATTGAATGCCGTGGTCTCGT
10419F	CGCACTCGACTACATTCCCA
10419R	GTGCAGAGATGCCCTTGACT
12762F	GTCAAAGAAGGGCAACCAGC
12762R	TGGTCTTCAGGACGAATCCAG

The number in the primer name is the same as the gene ID. For example, 12762F is used for amplification of FOXG_12762. All primers in this table were designed with a Tm of 60°C.

### ICL activity assay and measurement of succinic acid

Isocitrate lyase (ICL) activity was measured with an ICL activity assay kit of (Comin Corporation, Suzhou, China). After 24 h cultured in PDB, mycelia were collected and ground to a powder in liquid nitrogen. The mycelium powder was homogenized in 200 μl distilled water, and then centrifuged at 12,000 *g* at 4°C for 15 min. The supernatant was treated according to manufacturer’s protocol, and the absorbance of the samples at 340 nm was detected using a spectrophotometer (Agilent Biotek, Santa Clara, CA, USA). The ICL activity was expressed as nmol/min/g.

Succinic acid was detected by high performance liquid chromatography (HPLC). A RIGOL (Suzhou, China) L3000 chromatograph and RIGOL C18 reversed-phase column (250 mm × 4.6 mm, 5 μm) were employed. The mobile phase was prepared as follows: 1.56 g of sodium dihydrogen phosphate was dissolved in 800 ml of water, then 16 ml of methanol was added and the pH was adjusted to 4–5 with a phosphoric acid solution; 10 μl of samples were loaded; the flow rate was 0.8 ml/min; the column temperature was 30°C; the sampling time was 30 min at 214 nm UV.

### Luciferase reporter assay

The FOXG_12762 (NCBI Gene ID, mRNA accession XM_018392621) CDS region was inserted into the pEGFP-N3 (Takara Clontech, Kyoto, Japan) multiple cloning site (MCS) to construct our FoDbp40-EGFP fusion protein expression vector (vector 1). The FOXG_05529 (NCBI Gene ID, isocitrate lyase) promoter region (regarded as −2,000 to +200) was inserted upstream of *luc2* in pGL4.10 (Promega, Madison, WI, USA) to construct our 05529pro-luc2 expression vector (vector 2).

Human embryonic kidney 293 (HEK-293) cells were maintained in high-glucose, GlutaMAX™ Dulbecco’s Modified Eagle Medium (DMEM; Thermo Fischer Scientific, Waltham, MA, USA) supplemented with 10% heat-inactivated FBS (Gibco Thermo Fischer Scientific, Waltham, MA, USA) and cultured in 60 ml flasks kept at 37°C in a humidified incubator containing 5% CO_2_. For transfections, HEK-293 cells were grown in FBS-containing medium in six-well plates until they reached 70% confluency. The transfection solution was prepared by mixing vector 1, pGL4.10 (Promega, Madison, WI, USA) basic vector or vector 2, pGL4.74 (Promega, Madison, WI, USA) containing the luciferase reporter gene *hRluc* for internal reference, Lipofectamine® 3,000 (Invitrogen Thermo Fischer Scientific, Waltham, MA, USA). Twenty-four hours later fluorescence from the enhanced green fluorescent protein (EGFP) was examined under an Olympus Model IX71 fluorescent microscope (Evident Olympus, Tokyo, Japan) to judge whether the FoDbp40-EGFP fusion protein was successfully expressed. Firefly and *Renilla* luminescence were tested using a Dual-Glo® Luciferase Assay System (Promega, Madison, WI, USA)^2^ with a spectrophotometer (Agilent Biotek, Santa Clara, CA, USA). The relative expression of *luc2* was expressed as the ratio of firefly to *Renilla* luminescence signal. HEK-293 cells and pEGFP-N3 were obtained from Jilin University Mycology Research Center (Jilin, China). Primers used are provided in Supplementary Table S2.

### ATP level assay

Mycelia ATP levels were determined using an ATP assay kit (Beyotime, Jiangsu, China) according to the manufacturer’s instructions. After 24 h cultured in PDB, 100 mg of mycelia were collected and ground into powder in liquid nitrogen, the powder was homogenized in a lysis buffer and then centrifuged at 12,000 *g* for 5 min at 4°C. The supernatant was mixed with the working solution. The mixture was put into microwell plates and fluorescence intensity was measured with a spectrophotometer (Agilent Biotek, Santa Clara, CA, USA). The ATP levels were expressed as nmol/g.

### Western blot analysis

The western blot method was performed as previously described (Li et al., 2010). 1 × 10^6^ conidia were inoculated in 50 ml of potato dextrose broth (PDB) and cultured with shaking at 28°C for 24 h. Mycelia were harvested and ground into powder in liquid nitrogen, then suspended in radioimmunoprecipitation assay (RIPA) buffer containing 1 mM phenylmethylsulfonyl fluoride (PMSF). 20 μg of sample was loaded in each lane of a 10% SDS-PAGE gel. After electrophoresis, the samples were transferred to a polyvinylidene fluoride (PVDF) membrane. The membrane was then blocked with Tris-buffered saline with 0.1% Tween® 20 detergent (TBST) buffer containing 5% milk. After incubation with primary and secondary antibodies, blots were developed using enhanced chemiluminescence (ECL) western blot detection reagent (Bio-Rad, Hercules, California, USA) and images were acquired using a Tanon 4,200 Chemiluminescence Imaging System (Tanon, Shanghai, China). Antibodies used (anti-Actin, anti-AMPK alpha-1, anti-Phospho-AMPK alpha-1, mTOR, and anti-Phospho-mTOR) were purchased from Invitrogen (Thermo Fischer Scientific, Waltham, MA, USA).

### Statistical analysis

All statistical analyses were performed using GraphPad Prism software version 6 (Dotmatics, San Diego, CA, USA). One-way analysis of variance followed by *t*-test was used for comparisons between the groups. *p* < 0.05 was considered to indicate a statistically significant difference.

The flow chart of present study was provided in supplementary materials (Supplementary Figure S3).

## Results

### Screening of *Fusarium oxysporum* mutants with reduced growth

Mutants of *F. oxysporum* were obtained by random insertion of T-DNA into the *F. oxysporum* genome using ATMT. A single specific amplicon can be amplified from all mutants (Supplementary Figure S4). The sequenced fragment was 100% identical to the *neo* gene, which proved that the T-DNA was successfully inserted into the *F. oxysporum* genome.

**Figure 1:**
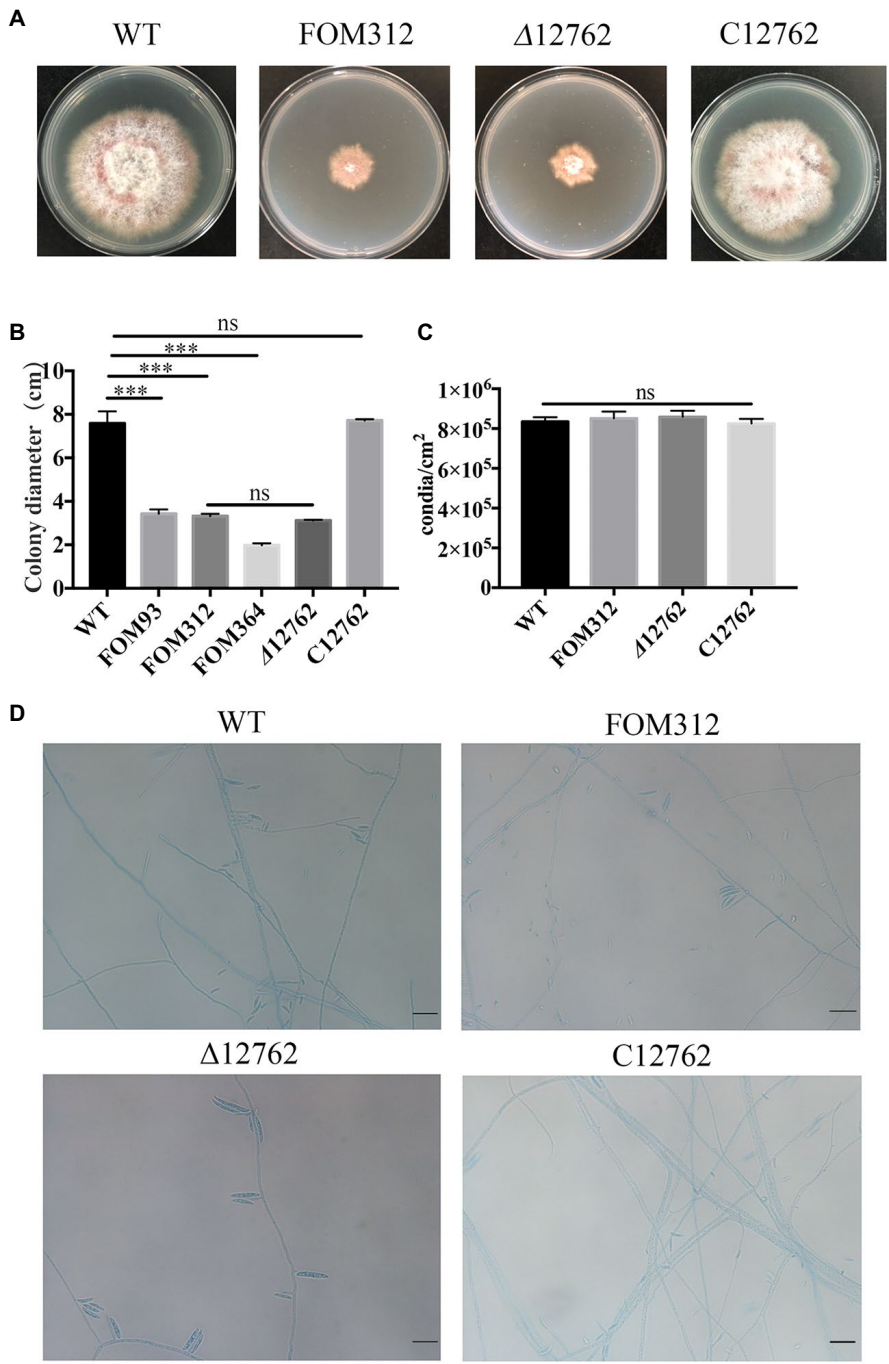
**(A)** Radial growth of wild type (WT), FOM312, *Δ*12762, and C12762. Strains were cultured on PDA medium and incubated at 25°C for 5 days. **(B)** The quantitative data for A (***p < 0.001). **(C)** Conidial production rate of wild type, FOM312, *Δ*12762, and C12762. **(D)** The slide culture of wild type, FOM312, *Δ*12762, and C12762 (bar = 25 μm). The experiment was repeated three times.

Mutant strains growth was compared to wild type *F. oxysporum* and FOM312 was identified with significantly reduced radial growth (Figure 1A). There were also other mutants were screened out with changed phenotype including slowed down growth, mycelial morphology changed, pigment decreased, etc., which were not discussed here.

### Detection of the expression of genes involved in glyoxylate metabolism pathway in FOM312

**Figure 2:**
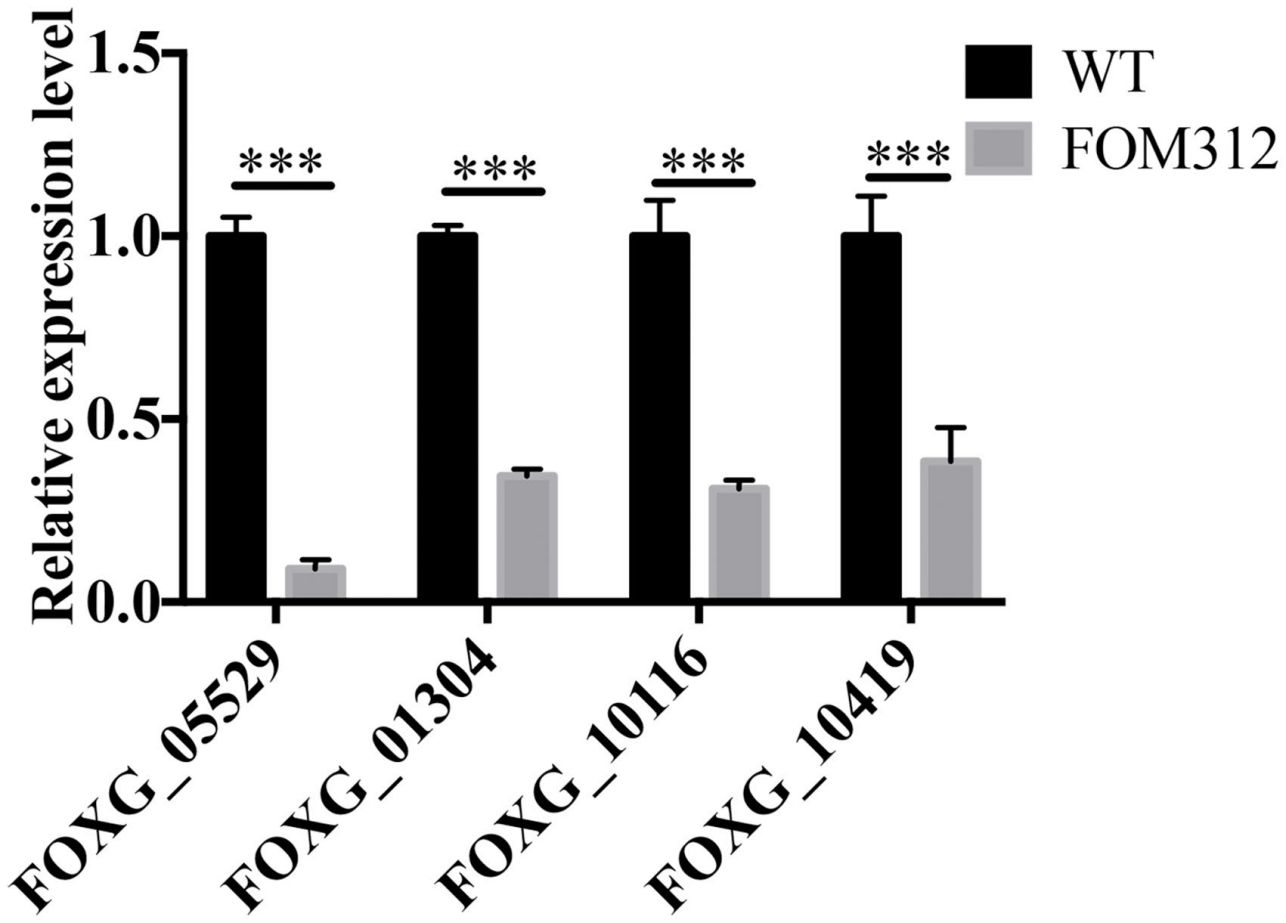
The relative expression level of four genes involved in glyoxylate metabolic pathway in FOM312. FOXG_05529 codes isocitrate lyase; FOXG_01304 codes 2-methylcitrate synthase; FOXG_10116 codes a formate/nitrite transporter domain; FOXG_10419 codes malate dehydrogenase. 18S rRNA was used as normalizing (***p < 0.001). The experiment was repeated three times.

The expression of four genes related to the glyoxylate metabolism pathway in FOM312 was detected by qPCR (Figure 2). The results showed that the expression of the four genes was down-regulated, and the expression of ICL was the most down-regulated. ICL is the rate limiting enzyme of the glyoxylate metabolism pathway. Therefore, we speculate that FoDbp40 may affect energy metabolism and the growth of *F. oxysporum* by regulating ICL expression.

### Analysis of T-DNA interrupted gene in the FOM312

T-DNA interrupted genes in FOM312 was verified by sequencing the TAIL-PCR products. The T-DNA in FOM312 inserted into FOXG_12762, which is located on chromosome 9 and encodes a hypothetical protein.

**Figure 3:**
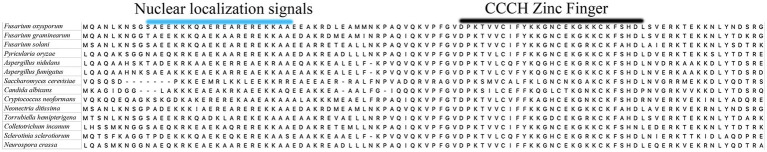
A part of the alignment result of amino acid sequences of FOXG_12762 and its most similar homologs from other fungi.

An amino acid sequence analysis performed with MEGA indicated that similar proteins are produced by other fungal species (Figure 3). FOXG_12762 encodes a hypothetical protein containing a CCCH zinc finger domain. This hypothetical protein has a high sequence identity (more than 70%) with homologs in common *Fusarium* species such as *F. graminearum* and *F. solani* and filamentous fungi such as *Aspergillus fumigatus* and *Torrubiella hemipterigena*. Sequence identity with other homologs, such as *Aspergillus nidulans* and *Aspergillus flavus*, is lower (60–70%). The homologs in *Aspergillus fumigatus* (79%), *Colletotrichum incanum* (80%), and *Torrubiella hemipterigena* (81%) are annotated as CCCH finger DNA binding proteins. According to the bioinformatic analysis for nuclear localization signals, there are three sections of the sequence predicted to be nuclear localization signals (Figure 3; Supplementary Figure S5).

### Constructs for gene deletion and mutant complementation of FOXG_12762

**Figure 4:**
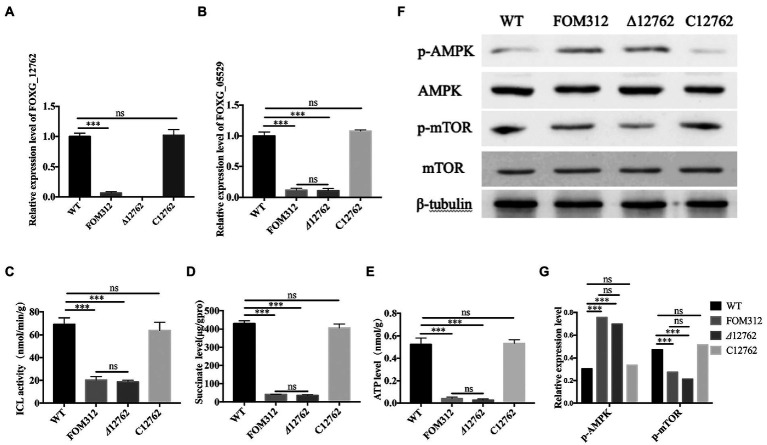
The detection of ICL-AMPK-mTOR axis. **(A)** Relative expression levels of FOXG_12762 in WT, FOM312, *Δ*12762 and C12762. **(B)** Relative expression levels of FOXG_05529 in WT, FOM312, *Δ*12762 and C12762. **(C)** ICL activity of WT, FOM312, *Δ*12762 and C12762. **(D)** Succinate level of WT, FOM312, *Δ*12762 and C12762. **(E)** ATP level in WT, FOM312, *Δ*12762 and C12762. **(F)** The expression level of AMPK, p-AMPK, mTOR, p-mTOR. **(G)** The quantization diagram of **E**, the expression of p-AMPK and p-mTOR was expressed as the ratio to β-tubullin (***p < 0.001). The experiment was repeated three times.

A knockout strain (*Δ*12762) and complementation strain (C12762) of FOXG_12762 were constructed. The deletion and complementation were verified by PCR (Supplementary Figures S1, S2). After 5 days of culture at 25°C, the *Δ*12762 colony was similarly sized to FOM312. The C12762 colony was similar in size to the wild type (Figures 1A,B). The expression of FOXG_12762 was significantly decreased after interrupted by T-DNA in FOM312, and was similar with wild type in C12762, while no signal was detected in *Δ*12762, which proved the successful deletion and complementation (Figure 4A). Based on the microscopic phenotype, the hyphae in the FOM312 and *Δ*12762 reduced compared with wild-type and C12762 (Figure 1D). Nevertheless, there was no much difference in conidial production between them (Figure 1C).

### Analysis of ICL expression regulation by FOXG_12762

The expression level of FOXG_05529 and ICL activity were detected and results show that the mRNA level of FOXG_05529 and ICL activity in *Δ*12762 and FOM312 decreased compared with wild type and C12762 (Figures 4B,C). HPLC results show that the level of succinic acid in FOM312 and *Δ*12762 also decreased, and the level of succinic acid in C12762 was close to that of wild type (Figure 4D). These results indicate that FoDbp40 can regulate the expression level and activity of ICL and affect the growth of *F. oxysporum*.

**Figure 5:**
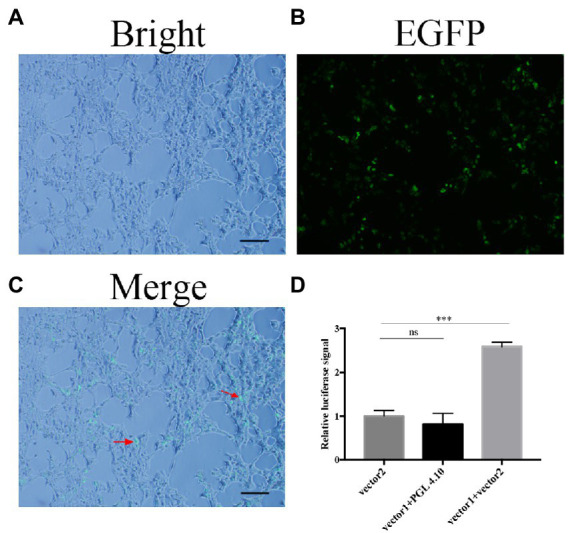
Transcription of FOXG_05529 is regulated by Fodbp40. **(A–C)** The FoDbp40-EGFP fusion protein was successfully expressed in HEK-293 (bar = 100 μm). **(D)** The expression level of 05529pro-luc2 in HEK-293 with and without FoDbp40 (***p < 0.001). The experiment was repeated three times.

FoDbp40 has high sequence identity to various CCCH zinc finger DNA-binding proteins in other fungi. This implies that it may has functions of binding to DNA and regulating transcription. To confirm whether FoDbp40 can regulate the transcription of the ICL-encoding gene FOXG_05529, the action of FoDbp40 on the promoter region of FOXG_05529 was investigated using dual luciferase reporter technology. The HEK-293 cells co-transfected with the FoDbp40-EGFP fusion protein expression vector (vector 1) and the 05529pro-luc2 expression vector (vector 2) can produce green fluorescence under 488 nm wavelength excitation, which indicates that the FoDbp40-EGFP fusion protein was successfully expressed in the HEK-293 cells (Figures 5A–C). Compared with the vector 2 transfection group, the *luc2* fluorescence signal of the vector 1 and vector 2 co-transfected group was significantly enhanced (Figure 5D). These results indicate that FoDbp40 can act on the FOXG_05529 promoter region to promote the transcription and expression of downstream genes.

**Figure 6:**
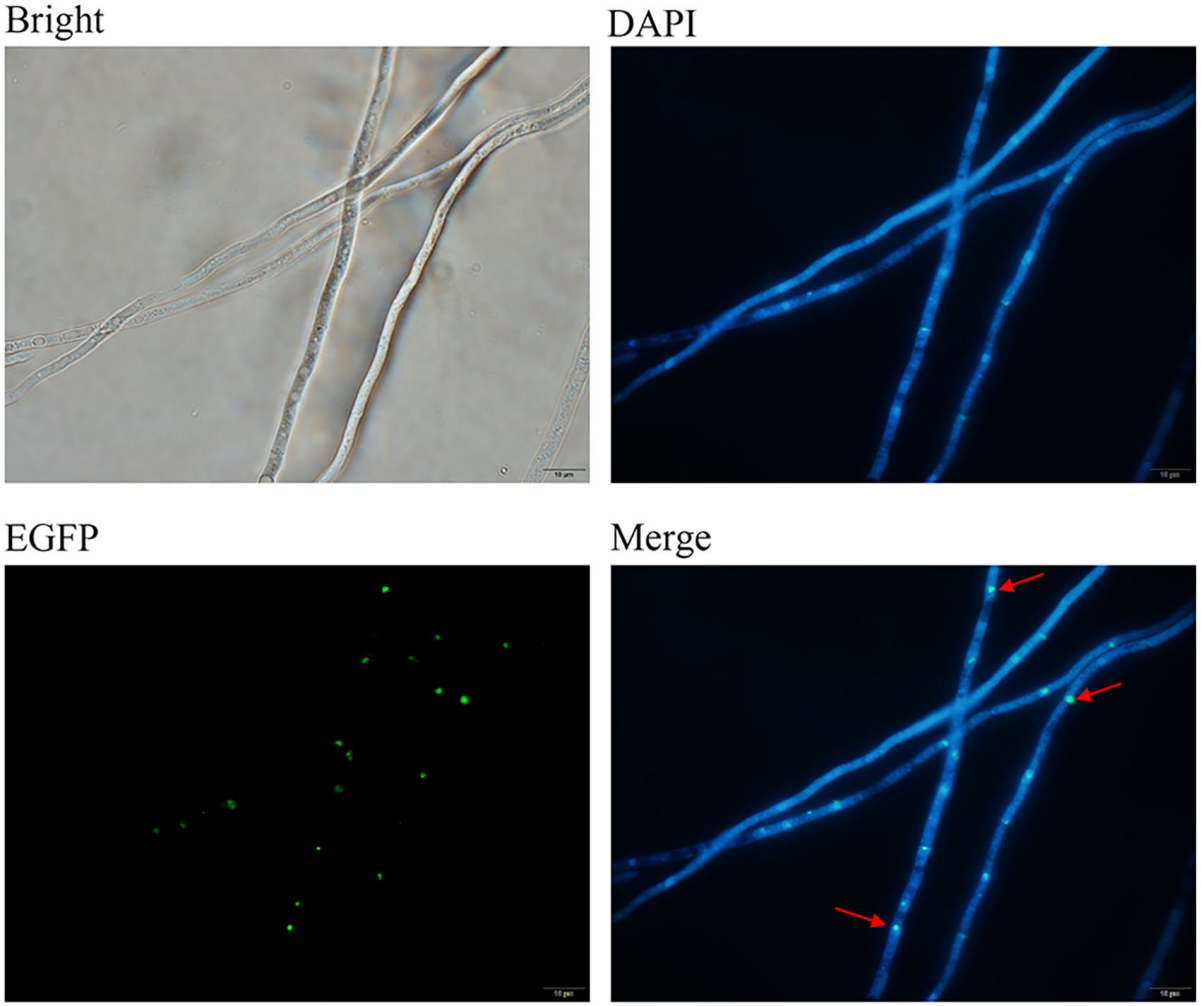
Subcellular localization of FoDbp40 in *F. oxysporum*. FoDbp40-EGFP is mainly localized in the nucleus, as demonstrated by 4′6-diamidino-2-phenylindole (DAPI) staining (bar = 10 μm).

The cellular localization of the FoDbp40-EGFP fusion protein was observed using fluorescence microscopy. Results show that FoDbp40-EGFP is primarily located in the nucleus, as demonstrated by DAPI staining (Figure 6).

### FoDbp40 regulates the AMPK/mTOR signaling pathway and energy levels

Considering the activity of ICL in regulating energy metabolism, we detected the ATP levels in wild type, FOM312, *Δ*12762, and C12762. ATP levels are decreased in the FOM312 and *Δ*12762 strains compared with the wild type and C12762 strain (Figure 4E). The 5′-adenosine monophosphate activated protein kinase (AMPK) and mammalian target of rapamycin (mTOR) phosphorylation levels were detected by western blot. The results show that the level of phosphorylated-AMPK (p-AMPK) increased and the level of phosphorylated-mTOR (p-mTOR) decreased in *Δ*12762 and FOM312 compared with wild type and C12762 (Figures 4F,G). These results indicate that the loss of FoDbp40 causes a decrease in ATP levels, which affects the regulation of AMPK/mTOR pathways, thereby causing reduced growth and virulence of *F. oxysporum*.

### Deletion of FOXG_12762 reduced the virulence of *Fusarium oxysporum*

**Figure 7:**
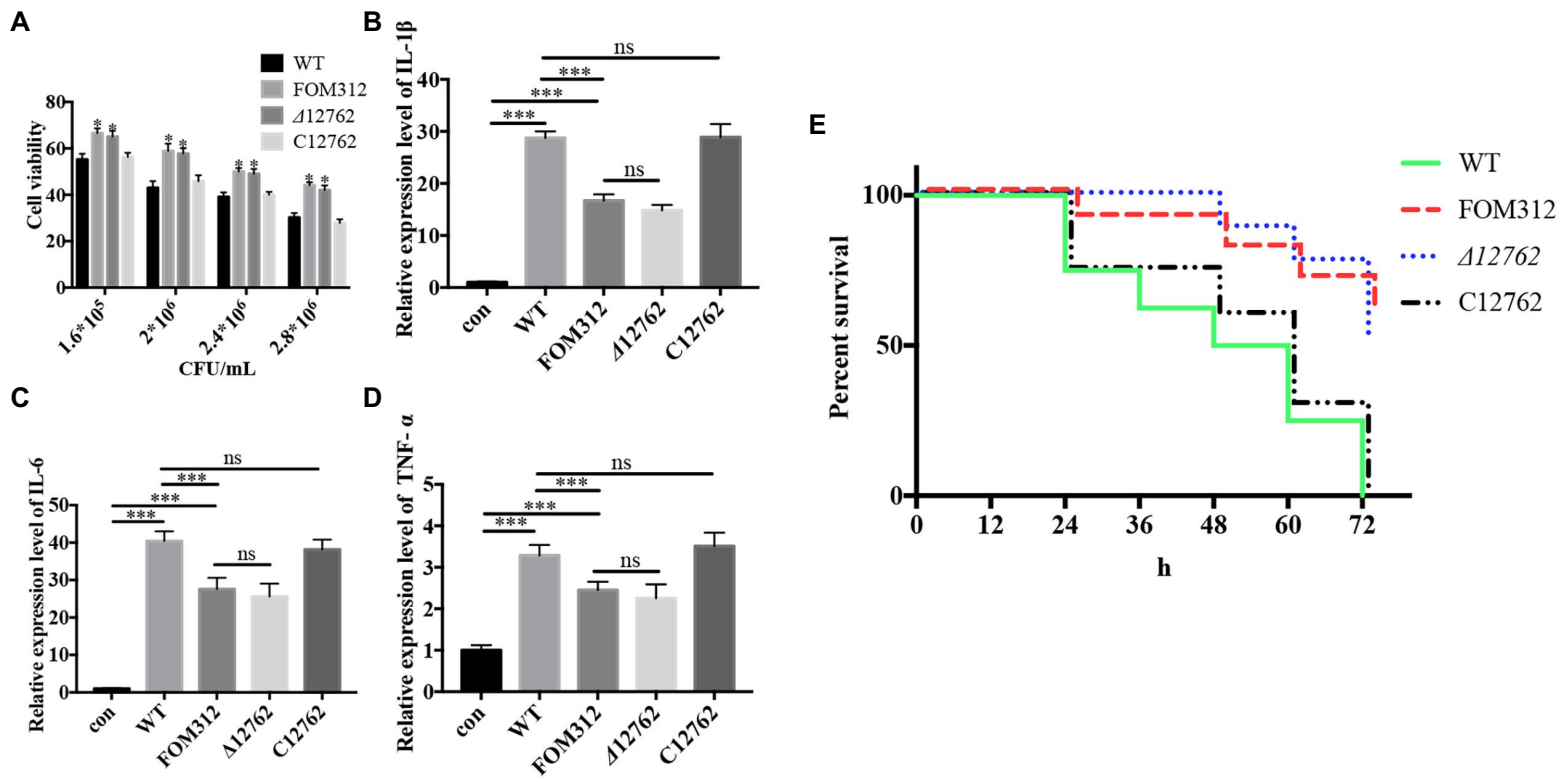
Virulence of *F. oxysporum*. **(A)** Cytotoxicity of *F. oxysporum* in HCEC (*p < 0.05 vs. wild type). **(B–D)** Expression of cytokines in HCEC co-cultured with *F. oxysporum* (***p < 0.001). **(E)** Virulence of *F. oxysporum* in zebrafish (*n* = 8). The experiment was repeated three times.

Different concentrations of conidia were co-cultured with HCEC for 24 h, and HCEC cell viability was detected by an LDH detection kit (Beyotime, Shanghai, China). Results show that FOM312 and *Δ*12762 (1.8 × 10^6^ CFU/ml for half maximal inhibitory concentration [IC_50_]) have a lower cytotoxicity compared with wild type (2.4 × 10^6^ CFU/ml for IC_50_). This indicates that the deletion of FOXG_12762 results in decreased *F. oxysporum* virulence in HCEC (Figure 7A).

The IC_50_ of wild type with HCEC was 1.8 × 10^6^ CFU/ml; therefore, this concentration was selected to stimulate HCEC cells to observe inflammation levels. Results show that the expression levels of IL-1β, IL-6, and TNF-α in HCEC increase after 6 h of stimulation (Figures 7B–D). Compared with the wild type treated group, the levels of inflammatory factors in the FOM312 and *Δ*12762 treated groups were lower, and the level of inflammatory factors in the C12762 treated group were close to those of the wild type treated group. This indicates that the deletion of FOXG_12762 can reduce the level of inflammatory response caused by *F. oxysporum*.

Zebrafish were inoculated with 1 × 10^4^ CFU/ml conidia to observe survival rates. Results show that the lethality of FOM312 and *Δ*12762 groups was lower than wild type and C12762, which suggest that the virulence of *F. oxysporum* decreased due to a deficiency of FOXG_12762 (Figure 7E).

## Discussion

*Fusarium* species are important plant pathogens and human opportunistic pathogens that seriously affect the yield of crops and human health. Fungi growth is closely related to virulence. Exploring the regulatory mechanism of *Fusarium* growth is helpful for the development of new drugs, as well as the prevention and control of *Fusarium* infection. Constructing random mutants of fungi by ATMT is convenient due to high efficiency, which has been extensively used in numerous fungi to clarify the function of unknown genes (Schmidpeter et al., 2017; de Vallée et al., 2019). We used the ATMT method combined with phenotypic screening to search for key genes in growth and regulatory mechanisms in *F. oxysporum*.

The glyoxylate metabolism pathway plays an important role in the growth, pathogenesis, and stress tolerance of fungi (Park et al., 2016; Vico et al., 2021). Therefore, the expression of four genes related to glyoxylate metabolic pathways were detected. The results showed that the four genes were down-regulated in FOM312 compared with wild type. Among the four genes, the rate limiting enzyme ICL was most down-regulated, so we speculated that the interruption of T-DNA in FOM312 may disturbed the expression of ICL.

In order to investigate the mechanism of regulation of ICL, the function of T-DNA interrupted gene FOXG_12762 in FOM312 was analyzed. FOXG_12762 encodes a hypothetical protein that we name FoDbp40. Presently the annotation of hypothetical proteins is primarily through homology search and the identification of conserved domains by sequence alignment algorithms (Ijaq et al., 2015). Our amino acid sequence alignment shows the FoDbp40 protein sequence to contain a CCCH zinc finger domain conserved in several pathogenic fungi (Figure 3). Homologs in other fungi such as *Aspergillus fumigatus* and *Torrubiella hemipterigena* are annotated as CCCH zinc finger DNA binding proteins. Although CCCH zinc finger proteins were known as RNA-binding proteins (Fu and Blackshear, 2017), Recent studies showed that CCCH zinc finger proteins also bind to DNA and modulate transcription (Zou et al., 2018; Wang et al., 2022). Therefore, we speculate that FoDbp40 may have the ability to bind target DNA and function in transcriptional regulation.

In this study, we observed the regulatory effect of FoDbp40 on ICL expression (Figure 4) and demonstrated that this regulatory effect is achieved at the transcriptional level by acting on the promoter region of ICL (Figure 5). Additionally, we observed the FoDbp40 protein to localize in the nucleus (Figure 6) in line with the bioinformatic analysis (Supplementary Figure S5), which is similar to other known CCCH zinc finger protein transcription factors, including C3H12 and SAW1 (Wang et al., 2020; Seok et al., 2022). Our results indicate that FoDbp40 is a novel transcriptional regulator that can affect the expression and activity of ICL.

The glyoxylate metabolic pathway saves carbon sources by skipping the step of generating CO_2_ in the tricarboxylic acid cycle (TCA) while generating required intermediates. This plays an important role in the regulation of ATP synthesis (Park et al., 2016). The expression and activity of ICL and corresponding succinate levels are decreased after the deletion of FOXG_12762 (Figure 4). These results demonstrated the regulatory effect of FoDbp40 on ICL and glyoxylate metabolic pathway.

As mentioned above, ICL is a key enzyme in the glyoxylate metabolism pathway, thereby regulating carbon metabolism and ATP synthesis (Gengenbacher et al., 2010; Selinski and Scheibe, 2014). AMPK, an AMP-dependent protein kinase, is a key molecule in the regulation of biological energy metabolism. Intracellular energy level can regulate the phosphorylation of AMPK, which in turn regulates the Ras, ERK, mTOR, and other related pathways that crosstalk with AMPK, thus affecting cell growth (Hardie, 2014; Grahl et al., 2015). The regulation of growth by the AMPK/mTOR pathways has been reported in yeast (Forte et al., 2019), but has not been reported in *Fusarium* species.

The affect of FoDbp40 on the level of ATP and AMPK/mTOR pathway which involed in the growth regulation was analyzed at next. The results showed that the deletion of FoDbp40 results in a severe decrease of ATP levels in *F. oxysporum* (by more than 80%), while promoting phosphorylation of AMPK and dephosphorylation of mTOR (Figure 4). These results demonstrated the regulatory effects of FoDbp40 on energy metabolism and AMPK/mTOR pathways through the ICL.

*Fusarium* species often cause corneal infection in clinic. Some studies have used HCEC cells to establish *in vitro* model of cornea infection by pathogens such as *Fusarium solani* (Kolar et al., 2017). *F. solani* and *F. oxysporum* both belong to the genus *Fusarium*, which were the most predominant pathogenic *Fusarium* in clinic with similar infection and pathogenic patterns. Therefore, HCEC cells were used in this study to evaluate the virulence of *F. oxysporum*.

The pathogenesis of keratitis is often accompanied by an inflammatory response related to its prognosis (Matsumoto et al., 2005). Pattern recognition receptors on cell surfaces can regulate the expression of inflammatory cytokines after recognizing pathogens. These include IL-1 β, IL-6, and TNF-α; all cause inflammatory response (Yu et al., 2017). After the deletion of FOXG_12762, the cytotoxicity of *F. oxysporum* conidia in HCEC was attenuated, and the expression levels of pro-inflammatory cytokines in infectious keratitis decreased (Figure 7).

Dananjaya et al. (2017) have used zebrafish to evaluate the virulence of *F. oxysporum*. Considering that *F. oxysporum* often cause superficial infection in clinic, we established a model of *F. oxysporum* infection in zebrafish with the method of bathing referring to the virulence assay of Laanto et al. (2012) proceeded with *Flavobacterium columnare*. The results showed that there was a stable killing effect on zebrafish infected with *F. oxysporum*. Therefore, we believe that this model is suitable for evaluating the virulence of pathogenic fungi in superficial infection. In this study, the deletion of FOXG_12762 results in attenuated virulence of *F. oxysporum* in zebrafish (Figure 7). These results indicate that FOXG_12762 plays an important role in regulating *F. oxysporum* virulence.

In summary, our findings demonstrate that the gene encoding ICL is a key component affecting the growth of *F. oxysporum*. Furthermore, the putative protein FoDbp40 can regulate the expression of ICL at the transcriptional level, thereby affecting the level of ATP and the AMPK/mTOR pathways, and consequently regulate *F. oxysporum* growth and virulence. ICL and FoDbp40 have potential as new targets in the development of antifungal drugs.

## Data availability statement

The original contributions presented in the study are included in the article/Supplementary material, further inquiries can be directed to the corresponding author.

## Ethics statement

The animal study was reviewed and approved by the animal ethics committee of Jilin University (Jilin University, Changchun, China).

## Author contributions

DH and LW: conceptualization and design. BZ and YZ: methodology and experiments. SG: data analysis. BZ and DH: original manuscript. LW: review and editing. All authors contributed to the article and approved the submitted version.

## Funding

This study was supported by grants from the National Natural Science Foundation of China (81772162 and U1704283) and the Foundation of Jilin Education Committee (JJKH20211150KJ).

## Conflict of interest

SG was employed by Beijing ZhongKaiTianCheng Bio-technonogy Co. Ltd., Beijing, China.

The remaining authors declare that the research was conducted in the absence of any commercial or financial relationships that could be construed as a potential conflict of interest.

## Publisher’s note

All claims expressed in this article are solely those of the authors and do not necessarily represent those of their affiliated organizations, or those of the publisher, the editors and the reviewers. Any product that may be evaluated in this article, or claim that may be made by its manufacturer, is not guaranteed or endorsed by the publisher.

## Footnotes

^1^ https://blast.ncbi.nlm.nih.gov/Blast.cgi

^2^ https://www.promega.com.cn/products/luciferase-assays/

## Supplementary material

SUPPLEMENTARY FIGURE S1 **(A)** Mechanism of the deletion of FOXG_12762. **(B)** Amplification of neo fragment from the candidate strains. M, maker; lane 1, wild type; lane 2, pXEN plasmid, 3-5, different candidate strains. **(C)** Amplification of part of FOXG_12762 sequence from the candidate strains. M, maker; lane 1, water as blank; lane 2, wild type; lane 3-5, different candidate strains.

SUPPLEMENTARY FIGURE S2 **(A)** Mechanism of the complementation of FOXG_12762. **(B)** Amplification of FOXG_12762-EGFP sequence from the candidate strains. M, maker; lane 1, Δ12762; lane 2-4, different candidate strains. A fragment (~4500bp) could be obtainded from the candidates.

SUPPLEMENTARY FIGURE S3 The flow chart of analyzing the function of FoDbp40 on the growth and virulence of F. oxysporum.

SUPPLEMENTARY FIGURE S4 Amplification of neo fragment (~700bp) in some randomly selected mutants. M: Trans 2 K marker; lane1: water as blank; lane2: pXEN plasmid; lane3: wild-type F. oxysporum; lane4-16: different mutants.

SUPPLEMENTARY FIGURE S5 The prediction of nuclear localization signals in FOXG_12762. Three nuclear localization signals were predicted.
