# Supplementary figures and images for: Hypothetical protein FoDbp40 influences the growth and virulence of Fusarium oxysporum by regulating the expression of isocitrate lyase
Source: Front Microbiol. 2022 Nov 28;13:1050637. doi: 10.3389/fmicb.2022.1050637 (PMC9742485; doi:10.3389/fmicb.2022.1050637)

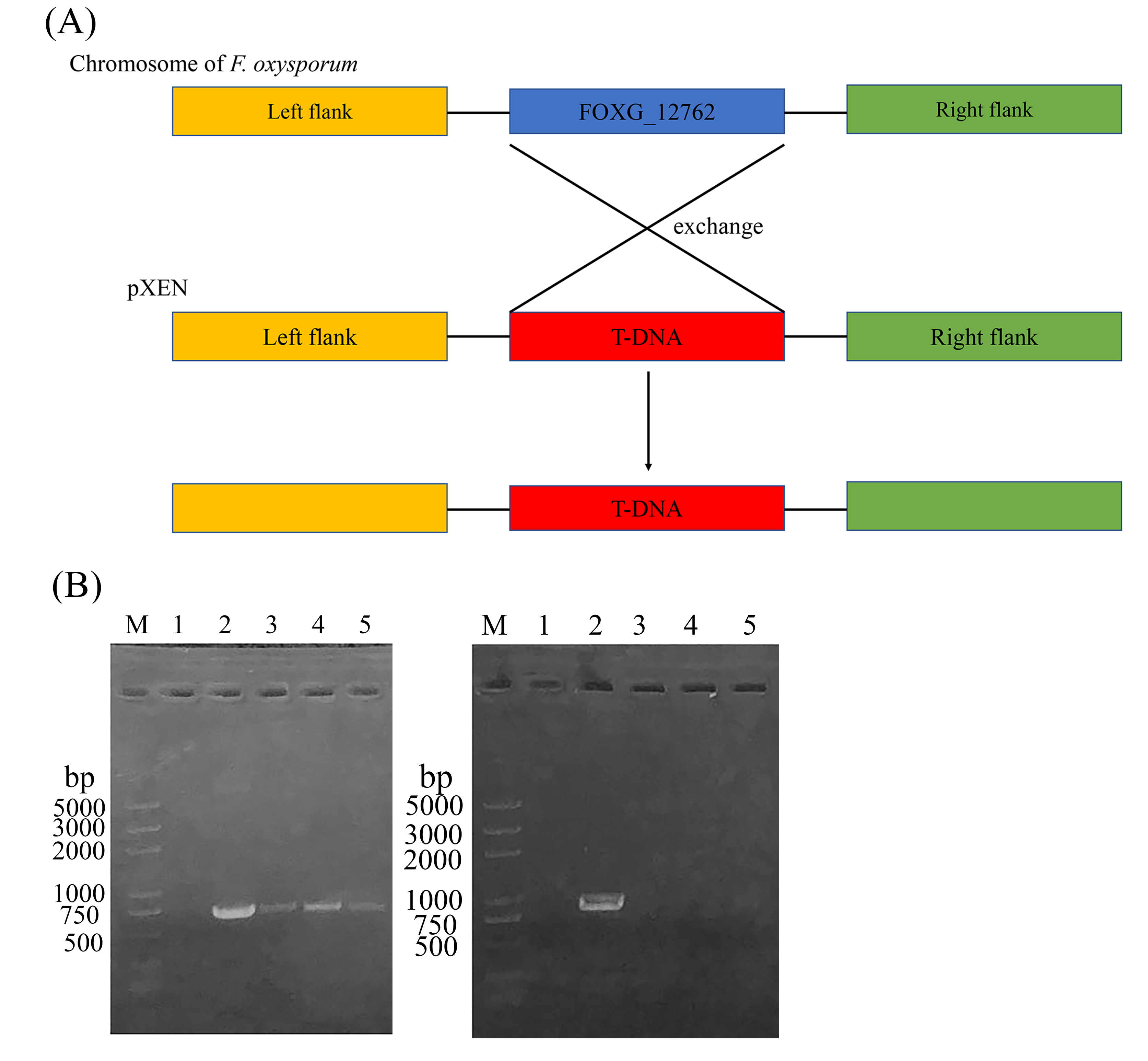

Supplement: SUPPLEMENTARY FIGURE S1 — (A) Mechanism of the deletion of FOXG_12762. (B) Amplification of neo fragment from the candidate strains. M, maker; lane 1, wild type; lane 2, pXEN plasmid, 3-5, different candidate strains. (C) Amplification of part of FOXG_12762 sequence from the candidate strains. M, maker; lane 1, water as blank; lane 2, wild type; lane 3-5, different candidate strains. [file Image_1.TIF]

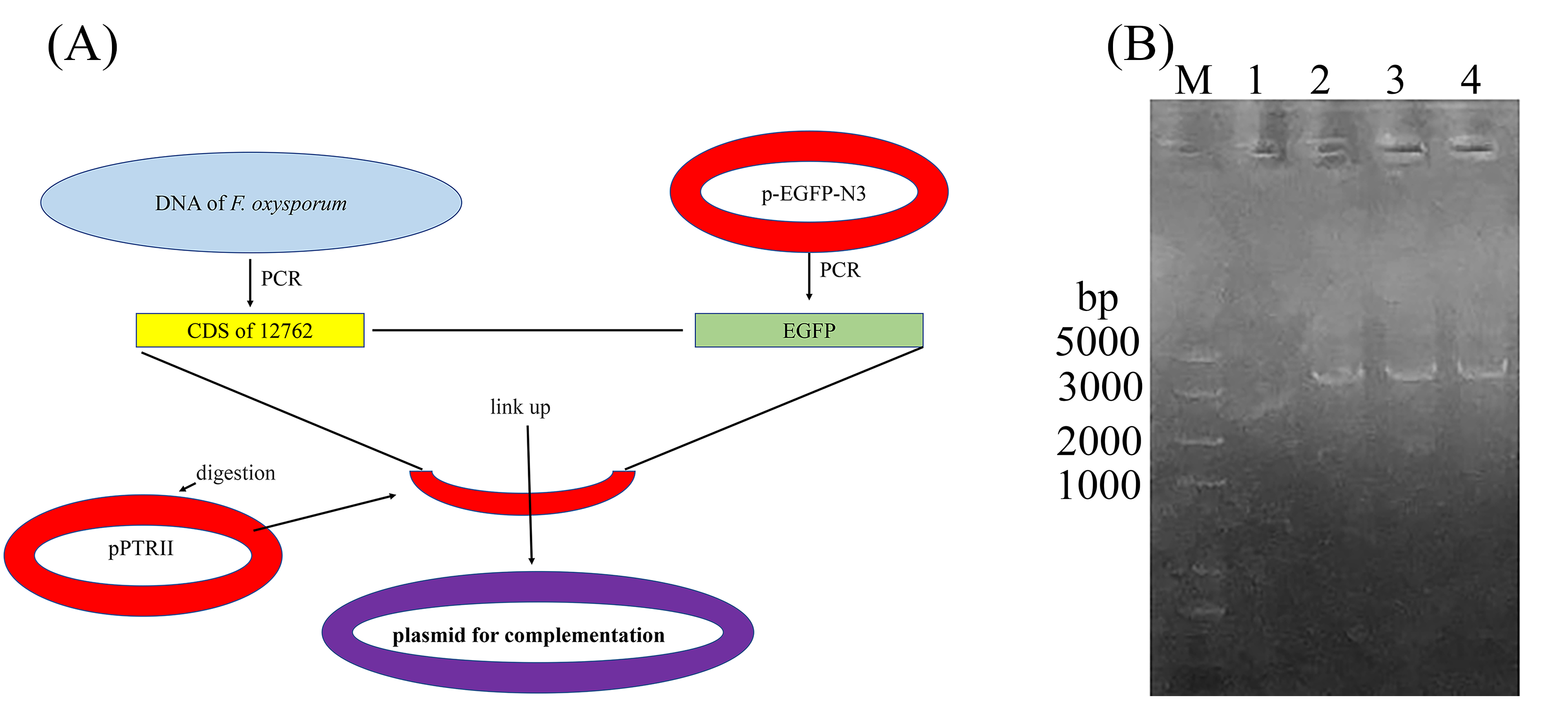

Supplement: SUPPLEMENTARY FIGURE S2 — (A) Mechanism of the complementation of FOXG_12762. (B) Amplification of FOXG_12762-EGFP sequence from the candidate strains. M, maker; lane 1, Δ12762; lane 2-4, different candidate strains. A fragment (~4500bp) could be obtainded from the candidates. [file Image_2.TIF]

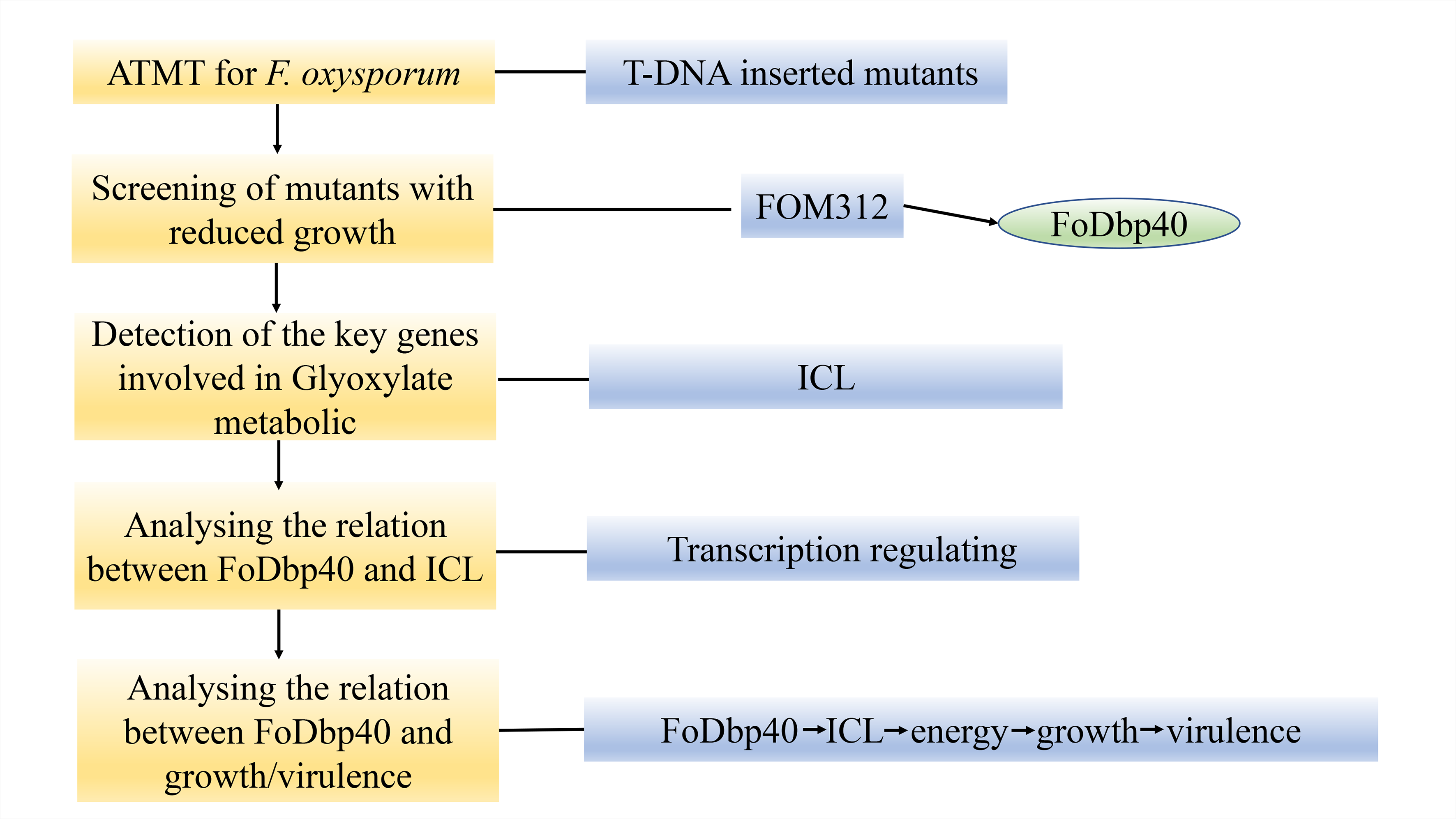

Supplement: SUPPLEMENTARY FIGURE S3 — The flow chart of analyzing the function of FoDbp40 on the growth and virulence of F. oxysporum. [file Image_3.TIF]

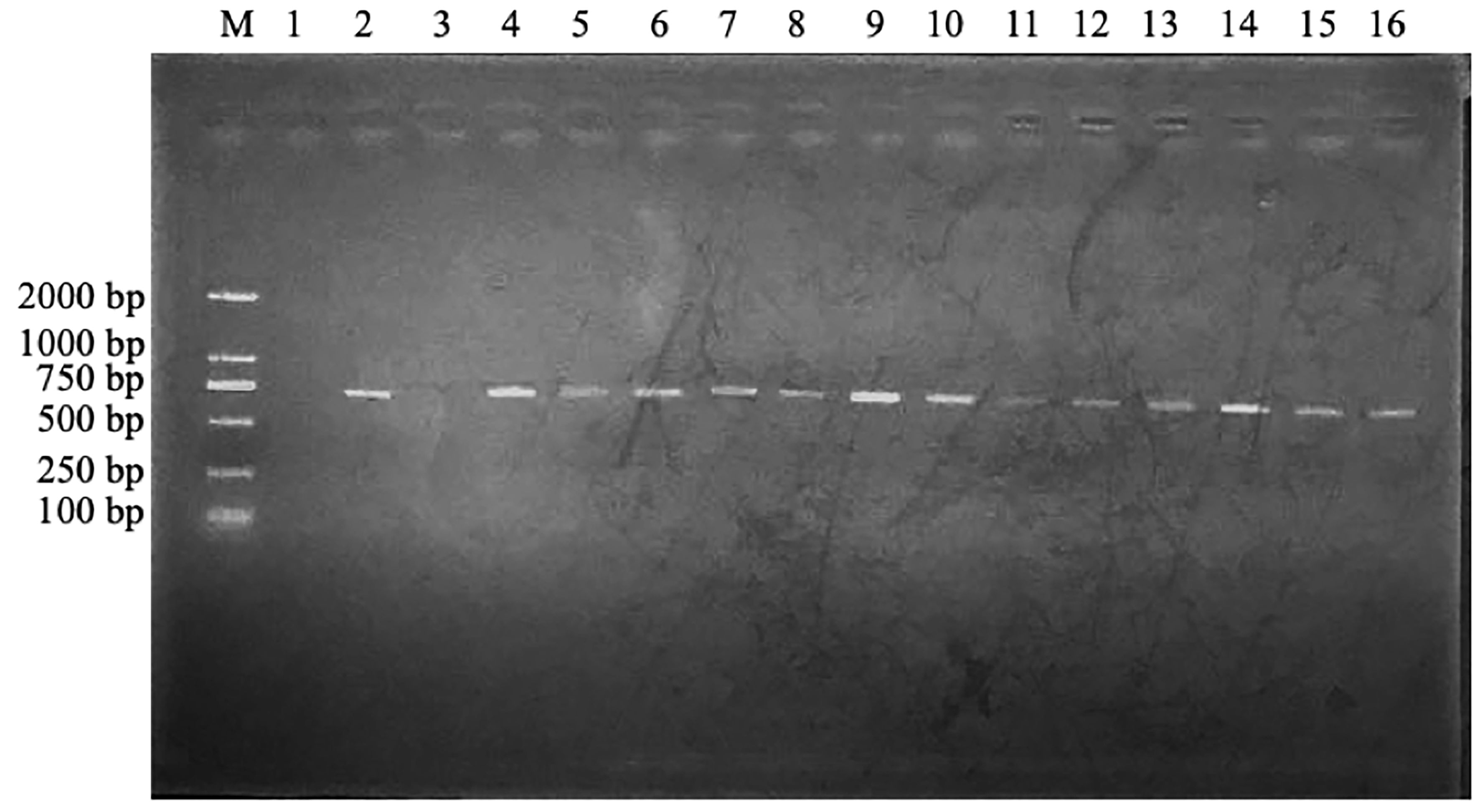

Supplement: SUPPLEMENTARY FIGURE S4 — Amplification of neo fragment (~700bp) in some randomly selected mutants. M: Trans 2 K marker; lane1: water as blank; lane2: pXEN plasmid; lane3: wild-type F. oxysporum; lane4-16: different mutants. [file Image_4.TIF]

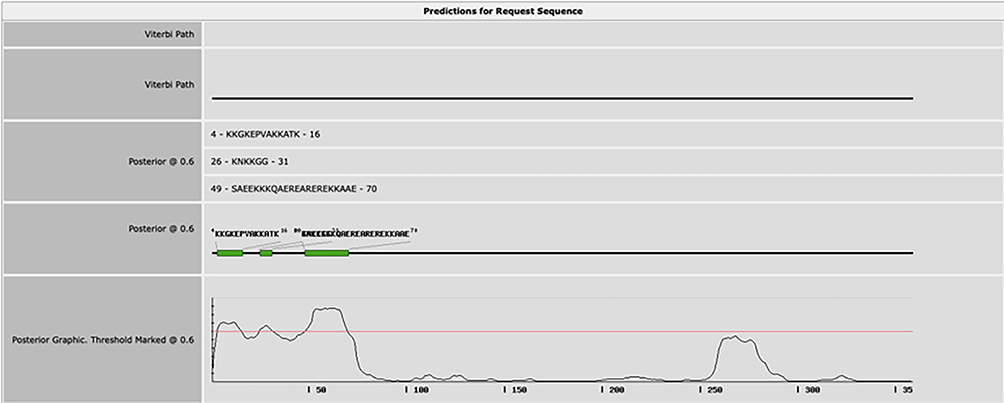

Supplement: SUPPLEMENTARY FIGURE S5 — The prediction of nuclear localization signals in FOXG_12762. Three nuclear localization signals were predicted. [file Image_5.TIF]
